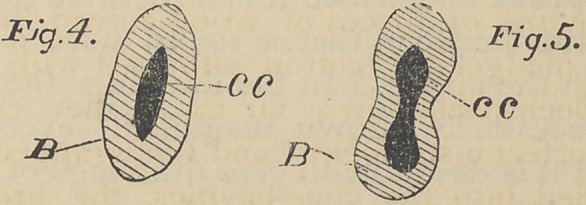# Apex Filling

**Published:** 1873-05

**Authors:** W. Storer How


### APEX FILLING.

The substance of two lectures delivered from the chair of Operative Dentistry, in the Ohio College of Dental Surgery, at the request of Prof. J. Taft, by W. Storer How, D. D S., Dec. 4th and 6th, 1872.

Every human tooth has within it one or more cavities normally filled with its pulps; and these cavities have a like configuration with the teeth; so that, represented by solid bodies, the cavities would appear as smaller teeth. The teeth themselves are difficult of exact description, because in situation, the top of a lower tooth corresponds to the bottom of an upper tooth; but as it is desirable that general terms should be employed in their descriptions, for the purposes of this lecture each tooth will be considered as "superior" in situation, and resting on its "crown" as a base, and to avoid confusion, the ordinary terms will be used when applicable ; it is however necessary for the proper understanding of the following methods and operations that the mind should always have before it the vertical position of the tooth as it rests upon its base. To this end simple outlines of the single, double, and triple rooted tooth, are now given, and are to be constantly borne in mind.

The usual anatomical description is "crown, neck, root, and pulp cavity." A glance at Fig. 1 will show that the form of its root is conical, having a base B. and an apex A; (for various reasons I have chosen *apex* rather than vertex or any other term;) it will also be seen that the roots of Figs. 2 and 3 may likewise be viewed as cones, with their bases at the necks of the teeth. As dentists we have at this time practically to do with the cavities rather than the roots, and I shall therefore further apply, and define the following terms:

Apex—The superior aperture of the cone cavity in each root.

Base—The inferior aperture of the cone cavity in each root.

Cone-cavity—The cone-shaped cavity in every root from *apex* to *base.*

Dental Cavity—The cavity within the crown of the tooth.

Accidental Cavity—Any other cavity in the crown or neck of the tooth, whether pathologically or artificially formed.

Our present purpose concerns the *accidental cavities* only, as a means of reaching the *cone cavities*, in order to fill them to their apices after the appropriate previous conditions shall have been fulfilled.

The treatment of alveolar abscess, the extirpation of the dental pulp, and the operation of "fang-filling," have occupied a prominent place in the practice, and in the writings of our profession, but as more than a general review of them does not come within the limits of this lecture, only incidental allusions can be made, to show what seem to have been errors or mistakes in theory and practice on the points named.

Alveolar abscess, or one variety of chronic odontalgia, resulting from the decomposition of the pulp, has been variously and remarkably treated; the primitive way being to extract the tooth; another, lately revived, was the removal of the tooth, excision of the sac, and replacement of the tooth; one way was to fill the dental and accidental cavities, and then drill through the root to the cone-cavity at its base, and thus drain off the pus as fast as formed in the sac; risodontripy, so-called, was a casual result of this sewerage operation. Extirpation of the pulps by direct excision, by the actual cautery, and by removal after obtunding its sensibility, have, with other similar means, been employed for the relief of acute odontalgia; and the common practice has been to fill the dental and accidental cavities, and after a year or more, relieve the inevitable chronic odontalgia by the previously described sewer opening or drain. Further attemps have been made to diminish the area of the concavities, and so to postpone the impending abscess for a longer time than usual by cone-shaped wires, or pieces of wood forced in and cut off at the bases; pledgets of cotton saturated with creosote, or iodine, have been packed in as far up as they could be carried with imperfect instruments; and foil wrapped on broaches has been carried into the cone-cavities, the broaches withdrawn, and repeatedly pushed back, with the effect of partially filling them for a short distance from their bases; the plastic compounds have also been used with like effects.

All these later methods, and the others not just now recalled, have this common and radical defect in *attempting only to reduce this capacity of the concavities to contain decomposed*

*matter*: the sewerage system being considered as only an expedient, and a base one at that. Indeed, the whole history of "fang filling," as hitherto written, shows that experiment in the line of expedients, without due process of reflection, has been the cause which has led to the several inefficient and indefensible operations of the present day; and that this statement is not too strong, will appear in the light of the theory and practice herein set forth, and descriptively termed *apex filling*.

Briefly stated, alveolar abscess is caused by decomposed matter within the cone-cavity, acting by a sort of distillation through the foramen at the apex, as an irritant to the investing tissues; therefore the reasonable process of treatment would be to remove the exciting cause, dry up the abscess through its natural sinus, the foramen, and then close that aperature by filling *first* the *apex* of the cone-cavity, then, if the investing tissues have been restored to their normal condition, there can be no recurrence of the abscess, because the *door is shut* by which the poisonous emanations found entrance into the healthy surrounding parts; and though the concavity should become a very pit of abominations, there could be no pernicious influence ever again exerted through that gate.

Having in the previous lecture determined what is necessary, and best to be done, let us now consider *how to do it*.

It was shown that a root divided in the plane of its axis, exhibited a hollow, which from its shape was termed a *cone-*

Fig. 4.

Fig. 5.

*cavity*; this description though not geometrically exact, is yet sufficiently so for the practical purposes in view, and is therefore not to be disturbed when the cross-section of a root shows the cone-cavity as an elipsoidal figure in either simple or complex form ; an illustration of the former being seen in Fig. 4, and of the latter in Fig. 5, which are respectively basic cross-sections of a cuspid, and a bicuspid root.

These two figures are types of all the cone-cavities at their bases, but the apices are always similar to Fig. 4 diminished in size; whether as in some bicuspids there is an attempt at two roots, or whether as in the lower molars two cone-cavities blend in a common apex.

It is of great importance that the shape of these cross-sections be carefully observed, as they have not been by those who have tried to fill them, by driving in and leaving those *round* tapering wires, though some operators taking the hint from the nerve instruments furnished by dental dealers, have endeavored to give their wires a shape proximating that of the cone-cavities to be filled; yet with the radical defect of both theory and practice, demonstrated in the previous lecture, namely, a reduction, and *only* a reduction of the mischievous capacity of the cone-cavity.

We have now reached several conclusions, which are of prime importance in relation to the subject under consideration, and they will be restated here, to bring them clearly before the mind in view of what is to follow.

First—The entire pulp is supposed to be removed, or the abscess to be completely cured, and the cone-cavity prepared for filling.

Second—The filling is to be *begun at the apex.*

It is obvious that the shape of the pluggers must conform to the shape of the cone-cavities, which we have seen to be conical, and ovoid, and which it is now to be said are generally of such small caliber, that a round wire can hardly be made to reach the apices. In the drawings the cone-cavities have been necessarily shown magnified several diameters, and now that it is desirable to give drawings of the pluggers, they too must be magnified, and as I turn to S. S. White's catalogue, to see what may be done by the engraver, I find on page 72, illustrations of "Dr. Hunter's pluggers," and on the opposite page, "Dr. Arrington's pluggers," which, while drawn to actual sizes, are really from three to four diameters too thick through the short axis of the cross-sections. I may well be pardoned a digression just here to say, that previous to the war I was associated with Dr. W. M. Hunter, and showed him this method of apex filling; and when after the war I came back to practice, I found these pluggers having a flattened oval cross-section, and of very coarse sizes for sale in the depots, as "Dr. Hunter's pluggers;" I was afterwards told that Mr. S. S. White saw them in his office, carried them off, and made them under the above title, without any word from him, Dr. Hunter, other than his permission to take them, and do what he pleased with them. I received them from my preceptor, Dr. Benjamin Lord, of New York, twenty years ago ; and to him, so far as known to me, is due credit for the original idea of apex filling, for the proper form of pluggers, for the method of conducting the operation, and for the barbed nerve-extractors, described on page 73 of the catalogue. For the extraction of nerves, I learned from Dr. Hunter, the use of a very fine five-sided broaches, annealed and hooked at the end, by passing across it a coarse file, and then the rotating of the broach in the cone-cavity cuts off, and removes the pulp.

It has been my part to carry the construction of the pluggers to a very fine point, by taking coarse annealed broaches, made up of the finest steel, filing them to a tapering square, then to a thin, flattened square, or paralellogram, then rounding the edges to a flattened oval, and finishing smooth with an old flat file. The broaches are then set in a steel socket, with soft solder, or tin, and are ready for use. Careful hammer-hardening improves them, but only the coarser ones will bear rough hammering, as the finer ones split under a heavy hammer; they are generally to be used without any temper, because of the liability of such delicate instruments to break under the pressure necessarily brought to bear upon them.

We have now to consider the materials available for the filling, and the forms they are to be given for that purpose. From Dr. Lord I learned to use gold foil, cut in strips of from a quarter to a half inch wide, carefully folded to about a half line in width, which were carried singly with the pliers to the bases of the concavities, and then caught upon the square cut ends of the pluggers, and carried to the apices of the conecavities, the pluggers slightly withdrawn, and then forced up again, carrying more foil, which was thus packed densely into the mouths of the foramen, the process being repeated until the cone-cavities were filled *from their apices to their bases.*

Practically, however, there were many cone-cavities presenting themselves, the apices of which were inaccessible to the folded strips; and in difficult positions, the chances of catching the strip on the point of the plugger was about one in ten; these two obstructions I partially overcame by cutting gold foil of various numbers, from 4 to 30 into firm thread-like strips, and by introducing several strips at a time into the base of the cone-cavity. I found, however, that, despite the greatest care in operating, some coarse strips, or some fine ones corrugated, and stiffened by repeated efforts to find by touch the small bases which could not be seen, would choke the cone-cavities about half-way up, and could be forced no further by the delicate pluggers reaching to those points. Tin foil then occurred to me as meeting the requirements of the case, because it is soft, welds easily, and does not harden by being crinkled; practice confirmed the theory, and after many vacillations, because other things being equal, gold would be preferable as less oxidizable; yet experiencing many chokings with gold, I finally settled upon tin, and now would give a hundred dollars an ounce for this purpose if it could not be obtained at a less cost.

To prepare the tin for filling, select the toughest foil you can find, cut a sheet in two, lay one-half in your left hand lengthwise, bring the thumb down upon the end near the tip of the forefinger, put the blade of the foil-shears under that end across the finger tips, cut a thread, open the shears, and, keeping the left thumb pressing lightly on the foil, feed it steadily forward after each stroke of the shears. A little practice enables one to prepare the foil finely and quickly. Taking now a bundle of these fibres in the plugging forceps they are placed in the dental cavity, and successively carried on the points of the pluggers to the apices of the cone-cavities, as previously described; and the operation thus rationally and carefully initiated, may be completed or not without affecting the point to which I have sought to direct your earnest attention, for the root filled at its apex cannot again induce aveolar abscess by the process of distillation, the mouth of the retort being sealed.

There remains only to be added an injunction to be present, persistent, and particular, in conducting the operation through its preparatory stages, and your reward will be found in an experience, which will enable you to subsequently say, "That tooth is filled at its apex, and I know it is saved past any fear of neuralgia," or "taking cold in it," or "disease at the root, or of having "to have it out," and for such other phrases as are commonly used to mask ignorance and incompetency in this highest *apex* branch of your profession, you will have no further occasion.

## Figures and Tables

**Fig. 1. Fig. 2. Fig. 3. f1:**
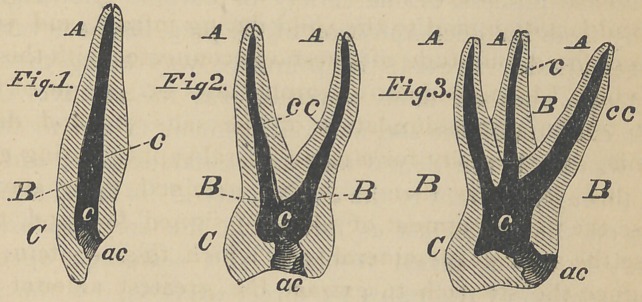


**Fig. 4. Fig. 5. f2:**